# Orbital intravascular papillary endothelial hyperplasia in a Nigerian child: a case report and review of the literature

**DOI:** 10.1186/1752-1947-6-300

**Published:** 2012-09-13

**Authors:** Oluyemi Fasina, Adewunmi Adeoye, Effiong Akang

**Affiliations:** 1 Department of Ophthalmology, University College Hospital, Ibadan, Nigeria; 2 Department of Pathology, University College Hospital, Ibadan, Nigeria

**Keywords:** Intravascular papillary endothelial hyperplasia, Masson’s hemangioma, Nigerian, Orbit, Proptosis

## Abstract

**Introduction:**

Intravascular papillary endothelial hyperplasia is a reactive proliferative lesion of endothelial cells in blood vessels. It typically presents as a painless, reddish purple lesion in the sites affected. The orbit remains an uncommon site of affectation of this relatively common disease. It is noteworthy that this is the first reported case, to the best of our knowledge, of orbital intravascular papillary endothelial hyperplasia in a Nigerian child.

**Case presentation:**

The case reported here is an orbital intravascular papillary endothelial hyperplasia causing non-axial proptosis and loss of vision in a 14-year-old Nigerian boy. We describe the clinical and histological findings of intravascular papillary endothelial hyperplasia in the orbit of this 14-year-old boy. The key distinguishing features are discussed and relevant literature is reviewed.

**Conclusion:**

Although unusual in presentation, intravascular papillary endothelial proliferation should be considered in the list of differentials of proptosis due to mass lesion in young Nigerians and, possibly, Africans.

## Introduction

Masson’s hemangioma was first described in 1923 by Pierre Masson as a locally occurring benign tumor in an ulcerated hemorrhoidal vein of a 68-year-old man [1]. He called it “Hémangioendothéliome Intravasculaire”, describing it as a form of tumor, due to proliferation of endothelial cells into the lumen of the vessel with subsequent obstruction and secondary degeneration and necrosis. It is a relatively common disorder and has been described by various names including Masson’s tumor, Masson’s hemangioma, Masson’s intravascular hemangioendothelioma, intravascular papillary endothelial hyperplasia (IPEH), and reactive papillary endothelial hyperplasia. However, it is now believed to be a reactive vascular proliferation following traumatic vascular stasis, and the current terminology, IPEH, was proposed by Clearkin and Enzinger [2] in 1976. It has previously been reported to occur in the extremities, the breast, the thyroid gland, the external jugular vein, on the tongue, the lip, within the oral cavity, as a neck mass, in paranasal sinus, intracranially and as a cause of facial nerve palsy [3-7]. However, in order of decreasing frequency, the lesion involves the fingers, head and neck, trunk, lower extremities and upper extremities [8]. Ocular involvement is unusual, and typically affects the eyelids [9,10]. In this article we report the clinical features, histopathology, and management of a solitary orbital lesion presenting with proptosis and poor vision in a 14-year-old Nigerian boy and a review of the relevant literature.

## Case presentation

An otherwise healthy 14-year-old Nigerian boy, of Yoruba ethnic group, presented to our Eye Clinic with a history of slowly progressive, painless right proptosis noticed shortly after birth. An increase in the rate of progression of the proptosis was noticed within the year of presentation and was associated with mild pain 2 weeks prior to presentation. The vision in his right eye had been poor since birth, and gradually worsened with the progressive proptosis. There were no complaints with his left eye. Specifically, he had no history of trauma, ear, nose and throat symptoms, swellings in other parts of the body, or weight loss. There was no significant medical history or history of allergy.

Ocular examination showed visual acuity of counting fingers at 2 meters in the right eye, about a 15mm non-axial proptosis that was inferolaterally displaced, palpable, mildly tender, and a superonasal, deep orbital mass, the posterior limit of which could not be delineated. There were no pulsations or bruit. Ocular motility was mildly restricted, with conjunctival injection, superficial punctate keratitis inferiorly, relative afferent papillary defect and clear lens. Fundoscopy revealed mild disc edema with blurred margins, but the vessels and macula were normal. The visual acuity in the patient’s left eye was 6/6, and intraocular pressures were normal in both eyes. Systemic examination was essentially normal.

**Figure 1:**
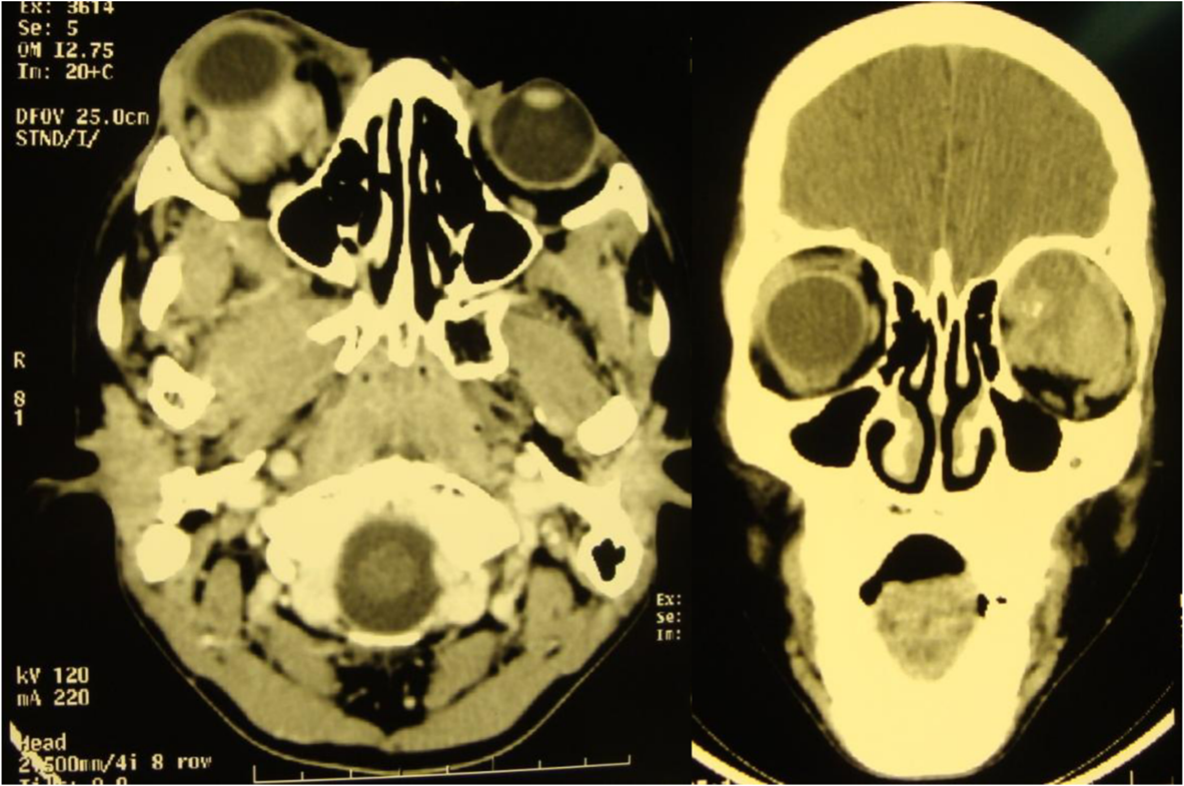
** Computed tomographic scan.** There is a superiorly located right orbital mass with retro-ocular extension and minimal enhancement with contrast.

An orbital computed tomography scan showed an isodense mass superiorly in the patient’s right orbit with retro-ocular extension and minimal enhancement with contrast. There was displacement of his optic nerve (Figure 1).

The patient subsequently had excision biopsy through a sub-brow incision where the mass was removed intact. The intra-operative finding was a well-encapsulated reddish brown mass measuring about 40×30×30mm in the superonasal orbit. He received systemic antibiotics and analgesics, and had an uneventful post-operative course.

**Figure 2:**
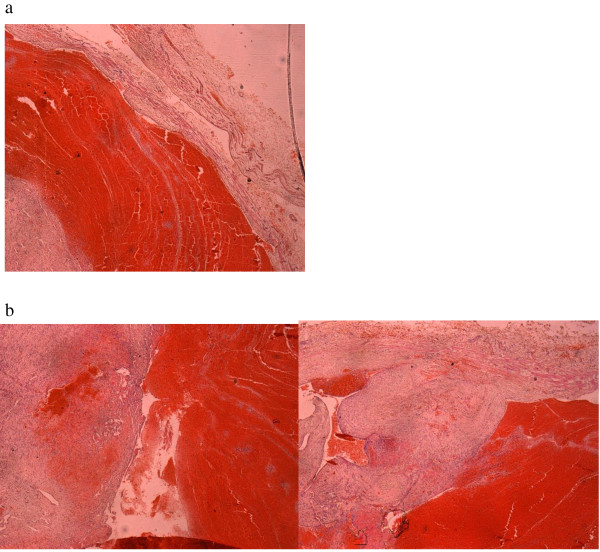
** a) Photomicrograph of the histology slide (hematoxylin and eosin (H&E) staining) of the lesion.** This shows the intravascular “tumor” with fibrin thrombus. The wall of the artery is seen to the right and the proliferation is to the left of the thrombus. **b**) Photomicrograph of the histology slide (H&E staining). Observation of the vascular mass shows proliferation of vascular channels within the lumen and the wall.

**Figure 3:**
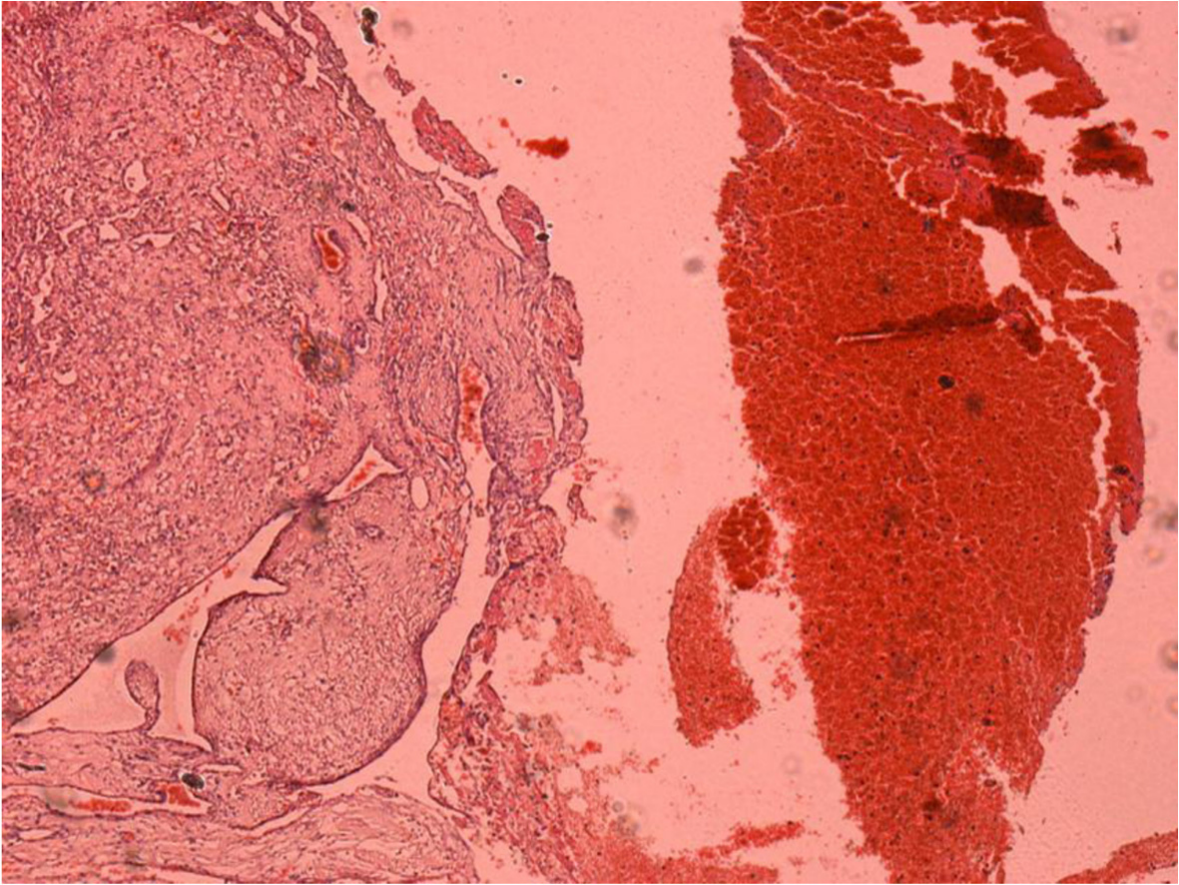
** Low-power view (**×**4) photomicrograph of the lesion.** This shows intravascular papillary proliferation of endothelial cells and associated stroma.

Histopathological examination of sections of the excised mass showed intravascular papillary proliferations of endothelial cells, with fibrin thrombi, causing partial occlusion of the lumen, with recanalization and extension into the vessel wall (Figures 2a, 2b and 3).

Post-operatively, the proptosis reduced to 3mm, however, mild restriction of ocular motility and poor vision persisted. There was no recurrence after 15 months of follow-up.

## Discussion

IPEH is a reactive proliferative lesion of endothelial cells in blood vessels. It is a relatively common condition constituting about 2% of the benign and malignant vascular tumors of the skin and subcutaneous tissues [11]. It seems to show no gender or age predilection because it has been reported in males and females ranging in ages from 9 months to 80 years. However, a female to male ratio of 1.2:1.0 with an average age of presentation of 34 years has also been reported [12]. Presentation of these lesions is mainly determined by their site of occurrence, most cases developing within vascular channels of the deep dermis or subcutis, and presenting as painless, reddish purple lesions, usually in the head and neck region and extremities [2,3]. Ocular involvement is unusual, and mostly involves the eyelids [8,9,13], whereas orbital lesions occur even less commonly [8,13,14].

IPEH had been suggested to represent either a primary endothelial proliferation with secondary thrombosis or vice versa. It was originally regarded by Masson [1] to be a true neoplasm due to endothelial proliferation and subsequent thrombus formation. However, it is now believed to be a reactive proliferation of endothelial cells that is associated with organization and recanalization of a thrombus [4]. The term IPEH, first given by Clearkin and Enzinger in 1976, [2] is now most widely used to describe the lesion. However, some authors [5] still consider IPEH to be a true neoplasm. Also, it has been proposed that IPEH formation is triggered off with release of basic fibroblast growth factor by the invading macrophages to the trauma site with proliferation of endothelial cells. Further release of more basic fibroblast growth factor by the proliferating endothelial cells occur, cascading into a positive feedback of endothelial cell proliferation [6].

Three clinical types of this lesion have been described. [3]. A primary or “pure” form arises within a normal blood vessel, most commonly a vein, and is often sited on the fingers, head and neck, and between the elbows and the hands. The secondary lesion or “mixed” form arises in the setting of a preexisting vascular malformation, such as a cavernous hemangioma or pyogenic granuloma and may be sited intramuscularly. The rarest type, an extravascular hemangioma, usually arises from an organizing hematoma. Our case could be considered to be a “mixed” form of IPEH arising on a background of cavernous hemangioma.

Histologically, IPEH consists of an intravascular proliferation of numerous papillae that are composed of a core of connective tissue and an endothelial surface. It can be distinguished from other neoplastic lesions because it is frequently well-circumscribed or encapsulated, with characteristic papillary fronds, and the proliferative process is entirely limited by the vascular wall. There is also an absence of features of malignancy such as mitotic figures, necrosis, nuclear pleomorphism, and infiltration into adjacent tissue [7]. The treatment for IPEH is surgical excision with complete resection as the goal; this is to forestall tumor recurrence in incompletely excised tumors [10]. Our patient underwent complete surgical excision with no recurrence of the tumor. However, his visual acuity did not improve post-operatively.

A major differential of IPEH is angiosarcoma. This however invades tissues outside the vascular channels and has more than one to two layers of endothelial cells covering the papillary formation. It also shows more malignant features on cytology [10].

## Conclusions

The first case of orbital involvement of IPEH was first described by Weber and Babel [14] in 1981. Since then there have been other reports [10,13] of orbital involvement. However, vision is usually preserved in these cases, at variance with our case, thus again re-emphasizing the need for public enlightenment on early presentation of patients with ophthalmic disorders in our region to prevent avoidable visual loss [15]. To the best of our knowledge our case is the first orbital Masson’s hemangioma reported in a Nigerian child.

In conclusion, we report a case of IPEH presenting as an orbital mass in a 14-year-old Nigerian boy. He underwent orbitotomy with complete tumor excision and had an uneventful post-operative period. Late presentation with attendant ocular morbidity is still a major challenge in our environment. Intravascular papillary endothelial proliferation should be considered a differential of proptosis due to mass lesion in young Nigerians and, possibly, Africans.

## Consent

Written informed consent was obtained from the patient’s father for publication of this case report and the accompanying images. A copy of the written consent is available for review by the Editor-in-Chief of this journal.

## Competing interest

The authors declare that they have no competing interests.

## Authors’ contributions

OF analyzed and interpreted the patient’s clinical data regarding the ophthalmic condition. AA performed the histological examination of the specimen, and was a contributor in writing the manuscript. EA participated in the histological examination of the specimen. All authors read and approved the final manuscript.
